# Implementation status of pharmacological studies in the development of orphan drugs

**DOI:** 10.1186/s13023-023-03000-0

**Published:** 2024-01-02

**Authors:** Saki Yokoshiki, Teruyo Arato

**Affiliations:** 1 Graduate School of Medicine, Hokkaido University, Kita 15, Nishi 7, Kita-ku, Sapporo, 060-8638 Japan; 2 Institute of Health Science Innovation for Medical Care (HELIOS), Hokkaido University Hospital, Kita 14, Nishi 5, Kita-ku, Sapporo, Hokkaido 060-8648 Japan

**Keywords:** Rare diseases, Orphan drugs, Non-clinical studies, Cell lines, Animal models, Biomarkers, Guidance

## Abstract

**Background:**

The nonclinical as well as clinical development of orphan drugs is difficult, owing to unknown pathophysiology and the absence of animal models. Both, the U.S. Food and Drug Administration (FDA) Guidance and European Medicines Agency (EMA) Guidelines, for orphan drug development describe non-clinical studies, but lack specific information, such as animal species and study design. Against this background, this study aimed to elucidate efficient methods for evaluating nonclinical efficacy based on a review report of orphan drugs approved in Japan.

**Results:**

A total of 184 orphan drugs, including 84 anticancer and 100 non-anticancer drugs, approved in Japan from January 2010 to December 2019 were investigated. Some anticancer drugs progressed to clinical development without distinct efficacy data in nonclinical studies. Patient-derived cells have been used for some drugs due to a lack of established cell lines. Cells used for non-clinical studies were devised for drugs indicated for cancers resistant to prior therapies, tumours with specific amino acid mutations in the target molecules, and solid tumours with specific biomarkers. For some non-anticancer drugs, similar disease animal models and normal animals were used for evaluation, since animal models did not exist. Biomarkers have been used specifically for evaluation in normal animals and as endpoints in some clinical trials.

**Conclusions:**

It was possible to evaluate drug efficacy by flexibly designing nonclinical studies according to disease characteristics for potentials orphan drugs. These approaches, which are not described in detail in the EMA Guideline or FDA Guidance, may thus lead to approval.

## Background

Recent advances in medicine have elucidated the pathophysiology of rare diseases, wherein identifying the aetiology and diagnosis are difficult, and new treatments have been developed. Drug development by pharmaceutical companies had focused on commonly occurring diseases; however, the rare diseases have recently gained considerable attention [1, 2]. Among the new drugs approved by the U.S. Food and Drug Administration (FDA) in 2020 and 2021, 60% are for rare diseases [3]. Currently, many of the drugs developed in academia are also for rare diseases. However, for developing orphan drugs, it is difficult to apply the clinical trial design used in general drug development because of the small number of patients. It is also difficult to evaluate drug efficacy in nonclinical studies, since the pathophysiology has not been fully elucidated [4].

Currently, no guidelines exist for orphan drug development in Japan. In 2006, the European Medicines Agency (EMA) issued Guideline on Clinical Trials in Small Populations [5]. The FDA also issued Rare Diseases: Common Issues in Drug Development Guidance for Industry in 2015 (revised in 2019) [6], and issued Investigational Enzyme Replacement Therapy Products: Nonclinical Assessment Guidance for Industry in 2019 [7]. In addition, orphan drug designation systems, trends in designated orphan drugs [8–11], and marketing approval statuses [8] have been reported. Factors to be considered for efficiently evaluating clinical efficacy for ultra-orphan drugs have been examined [12]. Current hindrances to the rapid development of safe and effective treatments for rare cancers have been discussed [13].

Non-clinical studies are important not only leading to clinical development but also for orphan designation. Both the FDA GUIDANCE and EMA GUIDELINE describe non-clinical studies but lack specific information, such as animal species and study design. In nonclinical development, animal models help to elucidate the aetiology and pathophysiology of diseases and are essential for the discovery and development of new treatments. In the case of rare diseases, the success rate of drug approval is low because of the poor predictive power of in vitro and in vivo models, which do not adequately model human drug responses [1, 14]. It is sometimes difficult to select appropriate animal models, which may not exist [1, 14]. Disease models of cancers, including rare cancers, have also been surveyed [11]. Research on animal models for rare diseases in several disease areas [15, 16]. However, the pharmacological studies designed for marketing approval applications, such as the cells and animals used for efficacy evaluation, have not been summarised.

Therefore, this study aimed to investigate the data packages and pharmacological study designs of approved orphan drugs and to clarify how efficacy evaluations lead to clinical development.

## Methods

We examined orphan drugs with new active ingredients and indications which were approved in Japan between January 2010 and December 2019. Review reports on these drugs were obtained from the Pharmaceuticals and Medical Devices Agency (PMDA) website (http://www.pmda.go.jp/PmdaSearch/iyakuSearch/). Data for nonclinical pharmacological studies for anticancer and non-anticancer drugs were examined separately. The results were considered complete when submitted as reference data. In anticancer drugs, we investigated the inhibitory effects on tumour growth in non-clinical studies, cancer cell types used for in vitro and in vivo studies, and their relationship with the inclusion criteria for clinical trials or approved indications. For non-anticancer drugs, we investigated the animal species and endpoints used for in vivo studies and the relationship between clinical and non-clinical endpoints.

## Results

In total, 184 orphan drugs were surveyed in this study. Eighty-four anticancer drugs included 47 drugs with new active ingredients and 37 drugs with new indications, while the 100 non-anticancer drugs included 57 drugs with new active ingredients and 43 drugs with new indications. At the time of approval in Japan, 124 of the 184 drugs were approved overseas.

## Anticancer drugs

### Implementation status

**Fig. 1:**
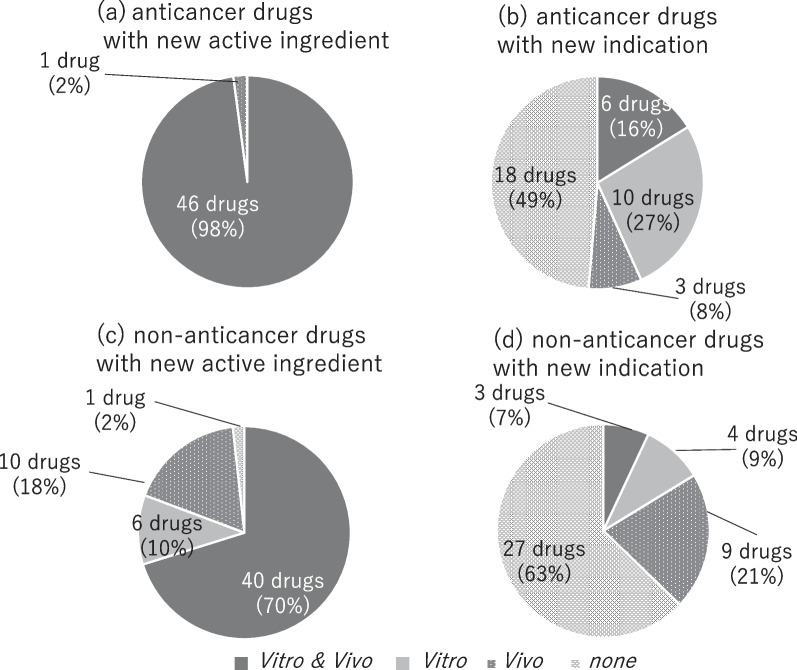
The implementation status of pharmacological studies

Both in vitro and in vivo pharmacological studies were conducted for 46 of the 47 anticancer orphan drugs with new active ingredients (Fig. 1). One drug, carmustine, was tested only in vivo. Of the 37 drugs with new indications, six were tested both in vitro and in vivo, 10 were tested only in vitro, three were tested only in vivo, and 18 were not tested, neither in vitro nor in vivo. However, for 15 of these 18 drugs, excluding imatinib mesylate (indication: hypereosinophilic syndrome, chronic eosinophilic leukemia), interferon gamma-1a (indication: Sezary’s syndrome), and sorafenib tosilate (indication: thyroid cancer), pharmacological data for the target carcinoma were submitted at the time of initial approval.

### Efficacy was not clear in nonclinical studies

Some anti-cancer drugs were inconclusive for clinical use based on the results of nonclinical studies but progressed to clinical development. Of the 10 drugs with only in vitro studies, lenalidomide hydrate (indication: myelodysplastic syndrome (MDS)) [17] provided insufficient evidence to improve anaemia caused by MDS, even though an inhibiting effect on tumour cell proliferation and a promoting effect on the differentiation of haematopoietic stem cells into erythrocytes were observed in vitro. The remaining nine drugs were determined to be effective based on the in vitro study findings and their mechanisms of action.

In vivo studies were conducted; however, the target cancer cells were not treated with forodesine hydrochloride (indication: peripheral T-cell lymphoma) [18], romidepsin (indication: PTCL) [19], vorinostat (indication: cutaneous T-cell lymphoma), or avelumab (indication: Merkel cell carcinoma) [20] (Table 1). Forodesine hydrochloride did not inhibit in vivo graft-versus-host reaction and delayed-type hypersensitivity reaction. Even during the in vitro study, the tumour growth inhibitory effect was observed only in “T-cell acute lymphoblastic leukaemia cell line” among untargeted cancer cells. Its efficacy in targeted cancer was explained by its mechanism of action. Romidepsin and vorinostat were effective against untargeted tumour cells. The efficacy of vorinostat was confirmed in vitro using a CTCL-derived cell line and in vivo using a colorectal cancer-derived cell line. However, at the approval review stage in Japan, additional in vivo studies using cells derived from targeted CTCL were required, since they are unknown to suppress CTCL cell proliferation by the same mechanism as its action on colon cancer-derived cells. In contrast, there was no discussion of its efficacy in CTCL-derived cells in the European Uinon and the United States. For the immune checkpoint inhibitor avelumab (indication: Merkel cell carcinoma), an in vivo study using a Merkel cell carcinoma cell line was not conducted, but a growth-inhibitory effect was observed in vivo using a mouse colon cancer-derived MC38 cell line expressing Programmed cell Death ligand 1(PD-L1). For nivolumab [21], pembrolizumab [22], and ipilimumab [23] (indication: malignant melanoma), also immune checkpoint inhibitors, growth inhibitory effects were not observed in mice transplanted with the malignant melanoma-derived potentially immunogenic cell line, B16-F10 melanoma cells (Table 1). On the other hands, these have been shown to be effective in vivo tests using cells from other cancers with highly immunogenic or with higher PD-L1 expression.

**Table 1:** Drugs whose efficacy was not confirmed in non-clinical studies

Non-proprietary name	Indication	in vitro tumour growth inhibition			in vivo tumour growth inhibition		
		Target tumour	Non-target tumour		Target tumour	Non-target tumour	
			Non-effective	Effective		Non-effective	Effective
Lenalidomide hydrate	Myelodysplastic syndromes	–	–	Burkitt's lymphoma acute myeloid leukaemia	–	–	–
Forodesine hydrochloride	PTCL	–	CTCL B lymphoblastoid lymphoma Promyelocytic leukaemia Malignant melanoma Breast cancer Colon cancer Renal cell carcinoma Prostate cancer Epidermoid carcinoma	T–cell acute lymphoblastic leukaemia	–	GVH response delayed–type hypersensitivity response (No tumour growth inhibition)	–
Romidepsin	PTCL	–	–	Leukaemia Malignant lymphoma	–	–	Leukaemia Colorectal cancer Reticulum cell sarcoma Malignant melanoma Lung cancer Stomach cancer Breast cancer Renal cell carcinoma Prostatic cancer
Vorinostat	CTCL	〇	–	Colorectal cancer	–	–	Colorectal cancer
Avelumab	Merkel cell carcinoma	–	–	–	–	–	Colorectal cancer
Nivolumab	Malignant melanoma	–	–	–	×	–	Colon cancer Fibrosarcoma Plasma cell neoplasm Kidney cancer
Pembrolizumab	Malignant melanoma	–	–	–	×	–	Colorectal cancer
Ipilimumab	Malignant melanoma	–	–	–	×	–	Fibrosarcoma Colon cancer

〇: effective, ×: non effective, −: not tested

*CTCL* cutaneous T-cell lymphoma, *PTCL* peripheral T-cell lymphoma, *GVH* graft-versus-host

Drugs were determined to be inconclusive for clinical use based on the results of non-clinical studies

In vivo studies using target cancer cells were not conducted in some drugs, and in vivo growth inhibitory effects were not observed in target cancer cells in other drugs

### Cells used for efficacy assessment

#### Use of patient-derived cells

Established cell lines are generally used for efficacy evaluation; however, patient-derived cells were used for some drugs due to the lack of established cell lines. Only patient-derived cells were used for mogamulizumab (indication: C–C chemokine receptor type 4 (CCR4)-positive peripheral T-cell lymphoma (PTCL)), ofatumumab and ibrutinib (indication: chronic lymphocyte leukaemia). Both established cell lines and patient-derived cells were used for mogamulizumab (indication: CCR4-positive adult T-cell leukaemia) [24]. The antibody-dependent cellular cytotoxicity (ADCC) activity of mogamulizumab was 53–59% in human adult T-cell leukaemia (ATL)-derived cell lines and a human T-cell leukaemia virus type 1-derived cell line, and it was 17–64% in CD3-positive cells isolated from 10 patients with ATL.

#### Selection of cells by indication

To evaluate efficacy against target protein-positive cancers, target protein-positive cells were created by introducing a gene expressing the target molecule into mogamulizumab (indication: CCR4-positive adult T-cell leukaemia) [24]. CCR4 gene-transfected cell lines with different CCR4 protein expression levels were used in addition to CCR4-positive cell lines. ADCC activity increased depending on CCR4 protein expression levels.

Drugs for cancers that are resistant to prior therapies include ceritinib (indication: *ALK*-positive non-small cell lung cancer (NSCLC) resistant to crizotinib) [25] and ponatinib hydrochloride (indication: chronic myelogenous leukaemia resistant to prior therapy). For ceritinib, four crizotinib-resistant cell lines were used for in vitro studies, and three cell lines with two crizotinib-resistant mutations were used for in vivo studies. Patients with *ALK* fusion-positive NSCLC resistant to crizotinib were included in the ceritinib clinical trials. In ponatinib, cell lines with resistance mutations to specific drugs were not used, and the growth-inhibitory effect was examined in vitro using breakpoint cluster region-abelson (BCR-ABL) fusion gene-transfected cells with five types of point mutations. Ponatinib was effective against chronic myeloid leukaemia that is resistant to imatinib, dasatinib, nilotinib, and bosutinib for the following reasons.

1. The inhibitory effect of ponatinib against wild-type BCR-ABL was more potent than that of imatinib and nilotinib and comparable to that of dasatinib.
2. Only ponatinib inhibited the growth of BCR-ABL^T315I^-expressing tumours.

However, dasatinib and nilotinib were only considered as treatment-resistant drugs in the inclusion criteria for clinical trials.

Drugs approved for tumours with mutations in specific amino acids of target molecules were vemurafenib, dabrafenib mesylate [26], trametinib dimethyl sulfoxide [27], encorafenib [28], and binimetinib [29] for malignant melanoma, and dabrafenib mesylate [30] and trametinib dimethyl sulfoxide [31] for NSCLC. In the case of vemurafenib for malignant melanoma (Table 2), v-raf murine sarcoma viral oncogene homolog B (*BRAF*) various V600 mutants have been used to examine the inhibitory effects on kinase activity and phosphorylation. *BRAF* V600E and V600D mutant cell lines were used for in vitro growth inhibition studies, whereas only the *BRAF* V600E mutant was used for in vivo studies. The clinical trials targeted patients with malignant melanoma, wherein mutations were detected using the “Cobas^®^ *BRAF* V600 mutation detection kit” to primarily detect the *BRAF* V600E mutation. Exploratory identification of *BRAF* mutations revealed that approximately 90% patients had a V600E mutation. Vemurafenib is approved for the treatment of malignant melanoma with mutations detected using the Cobas *BRAF* V600 Mutation Detection Kit in Japan. For tumours with specific amino acid mutations, the growth inhibitory effects were evaluated, at least in vitro using cancer cells with target mutations.

**Table 2:** Vemurafenib development for malignant melanoma with mutations in specific amino acids

BRAF	in vitro			In vivo	Clinical trial (Phase III)		
	Kinase inhibition	Inhibition of cell phosphorylation	Growth inhibition		Inclusion criteria	Vemurafenib (n = 336)	Control (n = 337)
V600E mutation	〇	〇	〇	〇	〇	295(88%)	303(90%)
V600D mutation	〇	〇	〇		△^a^	0	1
V600K mutation	〇				△^a^	33(10%)	24(7%)
Other mutations	10 other V600 mutations	V600R mutation				0	1
Wild type	〇	〇	〇			1	1

Mutated cell lines used in non-clinical studies of cancer drugs with mutations in specific amino acids and inclusion criteria for clinical trials

Growth inhibitory effects were evaluated in vitro using cancer cells with target mutations

^a^The Cobas® *BRAF* V600 mutation test shows cross-reactivity to *BRAF* V600K and V600D

As drugs with indications for solid tumours with specific biomarkers, pembrolizumab [32] was approved for solid tumours with microsatellite instability (MSI-high) and entrectinib [33] for *NTRK* fusion gene-positive solid tumours. For entrectinib, various *NTRK* fusion-gene-positive tumour cell lines were used in vitro and in vivo (Table 3). The inclusion criteria for the clinical trial were “*NTRK* fusion gene-positive solid tumour”, regardless of tumour types. This trial included patients with sarcomas, NSCLC, and colorectal cancer. Since the *NTRK* fusion gene was a cancer driver gene and a certain response rate was observed by cancer type, it was approved for the indication of “*NTRK* fusion gene-positive solid tumours”.

**Table 3:** Target cancers in entrectinib development

Cancer with *NTRK* fusion gene expression	In vitro	In vivo	clinical trial (Phase II)		Approved indication
	Growth inhibition		Inclusion criteria	Enrolled n = 51	
Non-small cell lung cancer	〇	〇	〇	9 (17.6%)	〇
Colorectal cancer	〇	〇		3 (5.9%)	
Head and neck cancer		〇		0	
Sarcoma		〇		13 (25.5%)	
Other solid cancers				26 (51.1%)	

Cancer cell lines were used for tumour growth inhibition studies and cancer types in patients enrolled in clinical trials

The tumour growth-inhibitory effect has been studied using several cancer cell lines, and the inclusion criteria for clinical trials were not limited to specific tumours

### Non-anticancer drugs

#### Implementation status of non-clinical studies

Both in vitro and in vivo pharmacological studies were conducted on 40 of the 57 non-anticancer orphan drugs with new active ingredients (Fig. 1). The following six drugs were only tested in vitro: eculizumab, tafamidis meglumine, elosulfase alfa, dornase alfa, cysteamine tartrate, and letermovir. There were no animal models for eculizumab (indication: reduction of hemolysis in patients with paroxysmal nocturnal hemoglobinuria, monoclonal antibody against C5) [34], tafamidis meglumine (indication: delay of peripheral neurologic impairment in patients with transthyretin familial amyloid polyneuropathy, benzoxazole derivative) [35], or elosulfase alfa (indication: mucopolysaccharidosis type IVA, enzyme replacement therapy). The effect of dornase alfa (indication: alleviation of cystic fibrosis, DNA degrading enzyme) on sputum viscosity were only investigated in vitro, despite the existence of animal models. For cysteamine tartrate (indication: renal cystinosis), in vivo studies were not conducted since renal damage was not observed in the knockout mice. The remaining drug, letermovir (indication: prophylaxis of cytomegalovirus disease) [36] was an antiviral agent. Of the ten drugs tested only in vivo studies, six were prototype vaccines for pandemic influenza. In some recombinant protein products, such as velaglucerase alfa (indication: alleviation of symptoms of Gaucher disease, enzyme replacement therapy) and elapegademase (indication: adenosine deaminase deficiency, enzyme replacement therapy) [37], enzyme activity was measured as a release test, corresponding to an in vitro study. Metreleptin (indication: lipodystrophy, hormone replacement therapy) [38] was the only drug for which neither in vitro nor in vivo studies had been performed. This drug was presumed to be effective based on literature on the efficacy of leptin supplementation in animal models.

In case of additional indications, there were four drugs for which only in vitro studies were conducted. For two of these drugs, dried sulfonated human immunoglobulin (indication: Churg-Strauss syndrome and allergic granulomatous vasculitis) and propranolol hydrochloride (indication: infantile haemangioma, a non-selective beta-adrenergic receptor blocker) [39], there were no animal model. There were nine drugs for which only in vivo studies were performed, among which four were immunoglobulin preparations. Neither in vitro nor in vivo studies were conducted on 27 drugs. Eleven of these were monoclonal antibodies.

### Efficacy was not clear in nonclinical studies

The efficacy of some drugs was presumed based on their mechanism of action, although evidence of their effectiveness in nonclinical studies was limited. At the time of adding new indication (amyotrophic lateral sclerosis (ALS)) to edaravone, radical scavenger [40], there was no significant difference in most of the in vitro study endpoints, including the survival rate; however, efficacy was expected from the mechanism of action. Cases where the adequacy of the study design was uncertain included aminolevulinic acid hydrochloride (indication: visualisation of malignant glucose, a biological substance converted to protoporphyrin IX) [41] and metirosine (indication: catecholamine hypersecretion in pheochromocytomas, tyrosine hydroxylase inhibitor). For aminolevulinic acid hydrochloride, non-clinical studies using target malignant glioma cells was not conducted. Moreover, pretreatment in a clinical setting was not performed in the non-clinical study for metirosine.

### Animals used for in vivo studies

Of the 62 drugs for which in vivo studies were performed, only animal models were used for 42 drugs, both model and normal animals were used for 17 drugs, and only normal animals were used for the three drugs, eltrombopag olamine (indication: chronic idiopathic thrombocytopenic purpura, a thrombopoietin receptor agonist) [42], sodium phenylbutyrate (indication: urea cycle disorder, prodrug of phenylacetic acid), and icatibant acetate (indication: acute oedema attacks caused by hereditary angioedema, synthetic peptide with competitive antagonism of brazinikin B2 receptor). Eltrombopag olamine did not activate thrombopoietin receptor of all animal species except for chimpanzee, but no chimpanzee disease model is known. Therefore, normal chimpanzees were used for the pharmacological study. Some of the 17 drugs for which both model and normal animals were used, showed varying extent of effects between the two groups of animals. In case of lomitapide mesylate (indication: homozygous familial hypercholesterolemia, selective inhibitor of microsomal triglyceride transfer protein), the effective dose 50 (ED_50_) was 2.5 mg/kg in normal animals, but 0.15 mg/kg in animal models. However, many drugs showed more pronounced effects on healthy animals than those on animal models.

In some cases, animal models of similar diseases were used for evaluation as no animal models existed. Rufinamid (indication: seizure in Lennox-Gastaut syndrome, compound with triazole skeleton) has shown efficacy against various epileptic seizure animal models, e.g., axial tonic seizures, atonic and cataplectic attacks, atypical absence seizures, etc. although there is no animal model for Lennox-Gastaut syndrome. Based on these data, a phase III trial was conducted in patients with Lennox-Gastaut syndrome. To develop a paediatric formulation of bosentan hydrate (indication: pulmonary arterial hypertension (PAH), endothelin receptor antagonist) [43], a persistent pulmonary hypertension of the newborn (PPHN) model, wherein the foetal ductus arteriosus is ligated in the sheep uterus, was used rather than the PAH model. In the approval review, the efficacy against paediatric PAH could be estimated for the following reasons:

1. Pulmonary vascular resistance (PVR) of foetal sheep decreased.
2. The pharmacological effect on PAH was evaluated at the time of the initial application of adult PAH, considering that the pathology of increased PVR between adult and paediatric PAH as well as the mechanism of action were similar.

### Use of biomarkers

Biomarkers were specifically used for evaluation in normal animals, and some biomarkers were used as endpoints in clinical trials.

Biomarkers were used for efficacy evaluation in both animal studies and clinical trials for five drugs, four of which were metabolic drugs (Table 4). For example, betaine (indication: homocystinuria, reduces homocysteine by donating a methyl group) was evaluated using homocystine levels as the biomarker in normal animal studies, animal models, and clinical trials.

**Table 4:** Drugs evaluated in normal animals using biomarkers

Non-proprietary name	Indications		Endpoint in nonclinical pharmacological studies		Endpoint in clinical trials
			Normal animals	Animal models	
*Same biomarkers (normal animals, animal models, clinical trials)*					
Eltrombopag olamine^a^	Chronic idiopathic thrombocytopenic purpura	Biomarkers	Platelet count	–	Platelet count
		True endpoint	–	–	–
Romiplostim^a^	Chronic idiopathic thrombocytopenic purpura	Biomarkers	Platelet count	･Platelet count	･Platelet count
		True endpoint	–	–	–
Migalastat hydrochloride^a^	Fabry disease	Biomarkers	α-Gal A	α-Gal A GL-3	α-Gal A GL-3 eGFR
		True endpoint	–	–	–
betaine^a^	Homocystinuria	Biomarkers	Homocysteine BHMT MS	Homocysteine	Homocysteine
		True endpoint	–	–	–
lomitapide mesilate^b^	Homozygous familial hypercholesterolemia	Biomarkers	VLDL-C + LDL-C HDL-C TG	VLDL-C + LDL-C HDL-C TG TC	LDL-C
		True endpoint	–	–	–
*True endpoints cannot be used in animals (normal and model)*					
Sodium phenylbutyrate^a^	Urea cycle disorders	Biomarkers	Excretion of nitrogen	–	Plasma ammonia
		True endpoint	–	–	Hyperammonaemia
Eliglustat tartrate^a^	Alleviation of symptoms of Gaucher disease	Biomarkers	Glucosylceramide levels	Glucosylceramide levels	–
		True endpoint	–	–	Achieving all the therapeutic goals below
Burosumab^a^	FGF23-related hypophosphatemic rickets osteomalacia	Biomarkers	Serum phosphorus Urinary phosphate Calcium	Serum phosphorus Calcium	Serum Phosphorus OV/BV
		True endpoint	–	–	RGI-C score
Ambrisentan^c^	Pulmonary arterial hypertension	Biomarkers	Blood pressure	Blood pressure	–
		True endpoint	–	–	6MWD
Riociguat^c^	Inoperable CTEPH or postoperative persistent or recurrent CTEPH	Biomarkers	Arterial pressure Coronary blood flow Heart rate	The right ventricular systolic pressure	–
		True endpoint	–	–	6MWD
Metyrosine^c^	Pheochromocytoma	Biomarkers	Noradrenaline Dopamine Serotonin	Blood pressure Systolic pressure Heart rate	–
		True endpoint	–	–	Patients with ≥ 50% decrease in urinary netamephrine 2 fraction
Icatibant acetate^d^	Acute oedema attacks caused by hereditary angioedema	Biomarkers	Blood pressure	–	–
		True endpoint	–	–	VAS
*True endpoints only assessed in disease (animal models and clinical trials)*					
Fingolimod hydrochloride^a^	Multiple sclerosis	Biomarkers	SlP1 receptor	–	–
		True endpoint	–	EAE	Recurrence rate Gd
Miglustat^a^	Niemann-Pick disease type C	Biomarkers	Ganglioside GM1	Ganglioside	–
		True endpoint	-	Atactic gait Survival rate	Eye movement speed
Tetrabenazine^e^	Involuntary choreatic movement caused by Huntington disease	Biomarkers	Dopamine Noradrenaline Serotonin	–	–
		True endpoint	–	Stereotypy Motor disturbances	TCS
Natalizumab^e^	Multiple sclerosis	Biomarkers	White blood cell	–	–
		True endpoint	–	Reducing the incidence of EAE Prevention of recurrent EAE	Incidence rate of focal
Apomorphine hydrochloride hydrate^e^	Parkinson disease	Biomarkers	dopamine 3,4-dihydroxyphenylacetic acid	–	–
		True endpoint	–	Increase in angular momentum	UPDRS partIII score
Vigabatrin^e^	Epilepsia nutans	Biomarkers	Enzyme inhibitory action GABA release action	–	–
		True endpoint	–	Spasm	Spasm
Rifaximin^f^	Hepatic encephalopathy of hyperammonaemia	Biomarkers	NH_3_	NH_3_	NH_3_
		True endpoint	–	Survival rate ED50 of gentamicin	PSE
Catridecacog^g^	XIII A-subunit deficiency	Biomarkers	Cross-linked protein	–	–
		True endpoint	–	Time to rebleeding	Rate of bleeding

*α-Gal A* α-galactosidases A, *BHMT* betaine-homocysteine methyltransferase, *CTEPH* chronic thromboembolic pulmonary hypertension, *EAE* experimental autoimmune encephalomyelitis, *eGFR* estimated glomerular filtration rate, *GABA* gamma amino butyric acid, *Gd* gadolinium, *GL-3* globotriaosylceramide, *HDL-C* high-density cholesterol, *LDL-C* low-density lipoprotein cholesterol, *MS* methionine synthase, *OV/BV* rate of change in osteroid volume/bone volume, *PSE* portal systemic encephalopathy index, *RGI-C* radiographic global impression of change, *SlP1* sphingosine-1-phosphate, *TC* total cholesterol, *TCS* involuntary choreatic movement score, *TG* triglyceride, *UPDRS* unified Parkinson’s disease rating scale, *VLDL-C* very low-density lipoprotein cholesterol, *6MWD* 6 min walk distance

Biomarkers have been used as endpoints for in vivo studies using normal animals, animal models and in clinical studies

^a^Metabolic

^b^Hyperlipidaemia

^c^Cardiovascular

^d^Allergy

^e^Central nerve

^f^Antibiotic

^g^Biological

Biomarkers were used for both normal and animal models for seven drugs, including three metabolic drugs and three cardiovascular drugs, because the true clinical endpoints were not available for evaluation in animals (Table 4). For example, the 6-min walk distance was used as the true endpoint in the clinical trials of ambrisentan (indication: PAH, endothelin receptor antagonist) and riociguat (indication: chronic thromboembolic hypertension, guanylyl cyclase activator) [44]; however, blood pressure was assessed as a biomarker in animal studies.

True endpoints were used for in vivo studies using animal models and clinical trials to evaluate improvements in pathology, but biomarkers were used for in vivo studies using normal animals that did not show pathology in eight drugs, half of which were central nervous system drugs. Spasm frequency was used as an endpoint in animal models and clinical trials for vigabatrin (indication: Pilepsia nutans, GABA-T inhibitor), but enzyme inhibition and gamma-aminobutyric acid release were evaluated in normal animals.

## Discussion

Non-clinical pharmacological studies were designed to understand the mechanism of action of a drug on disease pathology for proving a potential clinical benefit. Therefore, we investigated the implementation status and content of pharmacological studies for orphan drugs approved in Japan. In vitro and in vivo systems were designed to evaluate the efficacy in non-clinical studies. In some anticancer drugs, patient-derived cells were used owing to the lack of established cell lines. For tumours resistant to prior therapies or those with specific amino acid mutations, cell lines with resistant or target amino acid mutations were used for non-clinical evaluation. For solid tumours with specific biomarkers, in vitro studies were conducted using multiple carcinoma cell lines. Since there are no target disease animal models for non-anticancer drugs, their efficacies were evaluated using normal animals or similar disease animal models. The efficacies of some drugs were evaluated using biomarkers.

Several anticancer drugs, such as those for T-cell lymphoma and melanoma, are being developed, although non-clinical studies have shown no efficacy. In vivo studies using target tumour cell lines were not conducted for histone deacetylase (HDAC) inhibitors, such as romidepsin (indication: PTCL) and vorinostat (indication: CTCL), which showed tumour growth inhibitory activity using other tumour cell lines. It is reported that HDAC inhibitors are effective against cancer types which expressed high level of HDAC, other than the clinically indicated cancers [13, 45, 46].

For immune checkpoint inhibitors, tumour growth inhibitory effects were not observed in vivo using target tumour cells. It is sometimes difficult to evaluate the efficacy of drugs that target immune responses, including immune checkpoint inhibitors, because transplantation models cannot completely immunologically recapitulate human cancerous tissues [11]. Therefore, it was reasonable to clarify the growth inhibitory effect using mouse models transplanted with cell lines which were not the target carcinoma, but had sufficient immunogenicity, such as the immune checkpoint inhibitors examined in this study.

In addition to established cell lines, patient-derived cells were used for the in vitro studies of some rare cancers due to the lack of cell lines. It may be useful for estimating their efficacy. Recently, it is remarked that not only in vitro studies using patient-derived cells, but also patient-derived xenografts (PDX), in which tumour tissue is directly transplanted into mice [47]. PDX preserves the histological features of the original tumour and is useful for predicting treatment effects. Although PDX were not used in the drugs examined in this study, a PDX library has been created in Japan, and PDX may be used in non-clinical studies [48].

The development of animal models for rare diseases is more difficult than establishing cell lines, and many rare genetic diseases do not have desirable animal models [1]. It is estimated that there are approximately 7000 rare diseases, but only 57 animal models were used to apply for European Union Orphan Medicinal Product (OMP) designation in the therapeutic areas of metabolic, neuromuscular, and ophthalmic diseases [16]. The EMA's recommendation on elements required to support the medical plausibility and the assumption of significant benefit for an orphan designation states that “If in vitro evidence only is available at the time of the application, the relevance of the findings should be discussed in the context of the proposed condition” [49]. This implies that in vitro studies alone are considered acceptable. The FDA GUIDANCE also states that “sponsors can submit data from relevant in vitro models as supportive information” [6]. In this study, only in vitro studies were conducted for some drugs because of the lack of animal models or because animal models showed no clinical symptoms. Pharmacological studies could be omitted if the mechanism of action is clear and the effects could be estimated, such as with antibodies and enzyme preparations.

Normal animals were used for the in vivo studies of 20 drugs. It may be possible to estimate efficacy not only in animal models but also in normal animals in cases like biomarkers may be used as indicators of disease improvement. However, as with lomitapide mesilate and eliglustat (indication: alleviation of symptoms of Gaucher disease, substrate reduction therapy), the degree of efficacy may differ between normal animals and animal models.

Animal models of similar diseases may be used if no animal models exist. In vivo studies for rufinamide have been conducted on various seizures presented by patients, because it is necessary to appropriately match animal models with the clinical syndromes [15]. The efficacy in the bosentan hydrate paediatric preparation was evaluated by combining the results of the adult PAH model and PPHN model because of no paediatric model of PAH. Thus, even in the absence of animal models, these effects can be evaluated in a complementary manner by combining the results from several animal models.

Patient-derived induced pluripotent stem (iPS) cells are used for the discovery of new disease targets and drug screening, although they were not included in this study. iPS cells are generated from patients with specific diseases with known characteristics that can indefinitely repeat differentiation; therefore, they are expected to be a new disease model, in addition to human culture cell lines and animal models of disease [1]. In Japan, the Blood Law was revised in 2020, enabling the use of blood-derived iPS cells for drug research and development. Approximately 70 studies using disease-specific iPS cell studies for drug screening are underway until 2022, with the support of Japan Agency for Medical Research and Development (AMED), and are expected to increase progressively.

The use of biomarkers was divided into the following three cases: (a) the same biomarker was used in in vivo studies using normal and animal models and in clinical studies; (b) biomarkers were used only in animals (animal models or normal animals) because the true endpoint used in clinical studies cannot be used in animals; and (c) biomarkers were evaluated only in normal animals, as the true endpoint can be used only when the disease is present. The efficacy in normal animals was assessed using biomarkers. The FDA’s Guidance for Enzyme Replacement Therapy [7] states that “if biomarker improvements are correlated with functional improvements, studies using animal models would be useful.” However, biomarker assessment is considered useful when normal animals with no clinical symptoms are used. In addition to enzyme replacement therapy, biomarkers could be used in areas such as metabolic and central nervous system drugs. The FDA’s Guidance for Industry Expedited Programs for Serious Conditions—Drugs and Biologics also states that the FDA will consider using biomarkers in conjunction with other data. In fact, in vivo studies using biomarkers were performed, in addition to in vitro studies to conduct the clinical trials of some products, such as eltrombopag olamine. In the case of rare disease, due to the lack of precedent for drug development, the data summarising the surrogate endpoints including biomarker of past approvals provided by FDA [50] may be informative, not only for designing clinical studies but also for non-clinical studies.

Both the EMA GUIDELINE [5] and FDA GUIDANCE [6] state that non-clinical study data are important for the development of orphan drugs, but there are few specific descriptions of non-clinical study design. This study showed that it is possible to evaluate the drug efficacy devising cells for anticancer drugs, use normal animals and biomarkers for non-anticancer drugs, and flexibly design nonclinical studies according to disease characteristics for orphan drugs and lead approval and orphan designation. Study designs could be discussed with the regulatory agencies, who recognise the challenges in the rare disease field. However, having certain standards based on the characteristics of drugs will lead to efficient development. The devised evaluation method clarified in this study is expected to contribute to the promote orphan drug development.

This study was conducted based on review reports disclosed at the time of approval. Since information on drugs whose development was discontinued was unavailable, it was not possible to investigate what kind of nonclinical data were obtained at the start of the first-in-human studies. In this study, we clarified that some drugs were approved despite showing in efficacy in non-clinical studies, and normal animals and biomarkers were used for non-clinical efficacy evaluation. If failure cases can be collected, it may be possible to reinforce the results of this study, to determine if the devised non-clinical evaluation methods will ultimately lead to approval.

## Conclusions

During the development of orphan drugs, some drugs progressed to clinical development without clear efficacy in non-clinical studies, and cell lines and in vivo study designs were developed. For some anticancer drugs, patient-derived cells were used because of the lack of established cell lines. For drugs indicated for tumours resistant to prior therapies or tumours with specific amino acid mutations, a non-clinical evaluation was performed using cell lines with resistant mutations or target amino acid mutations. If the indication was solid tumours with specific biomarkers, multiple cancer cell types were used in vitro. Because there are no animal models of target diseases for any non-anticancer drugs, the efficacy of some drugs was evaluated using normal animals, animal models with similar diseases, or biomarkers as endpoints. Thus, it is possible to evaluate drug efficacy using an evaluation system that is not specified in the EMA GUIDELINE or FDA GUIDANCE. The results of this study will greatly contribute to the development of orphan drugs.

## Data Availability

Not applicable.

## Abbreviations

ADCC: Antibody-dependent cellular cytotoxicity
ALS: Amyotrophic lateral sclerosis
ATL: Adult T-cell leukaemia
CTCL: Cutaneous T-cell lymphoma
EMA: European medicines agency
FDA: U.S. food and drug administration
HDAC: Histone deacetylase
HEL: Human erythroleukemic
iPS: Induced pluripotent stem
MDS: Myelodysplastic syndrome
MSI: Microsatellite instability
NSCLC: Non-small cell lung cancer
PAH: Pulmonary arterial hypertension
PPHN: Persistent pulmonary hypertension of the newborn
PVR: Pulmonary vascular resistance
